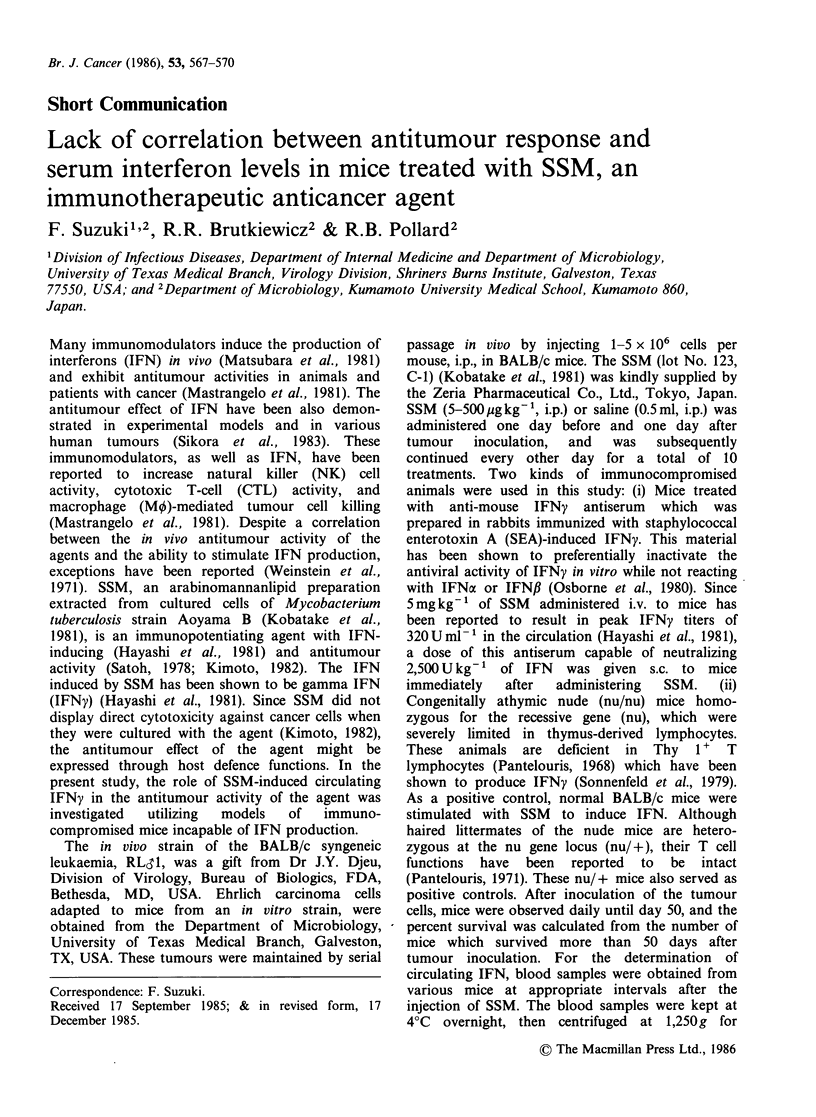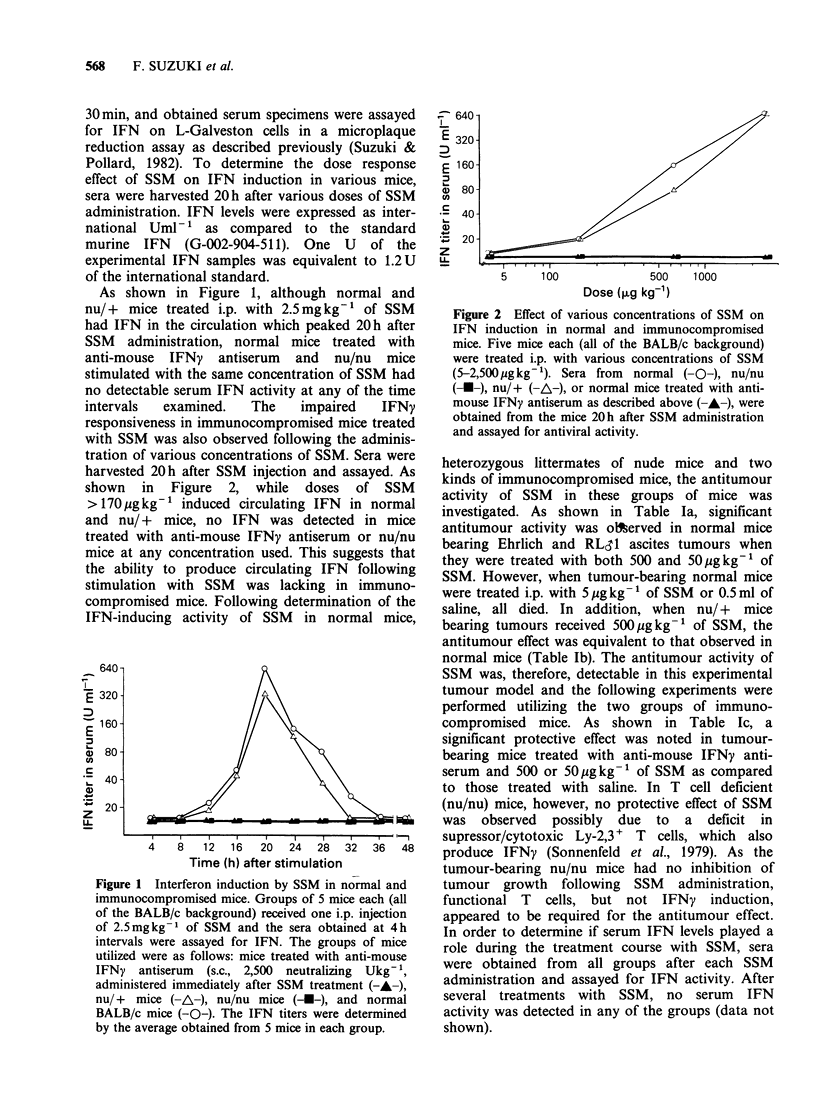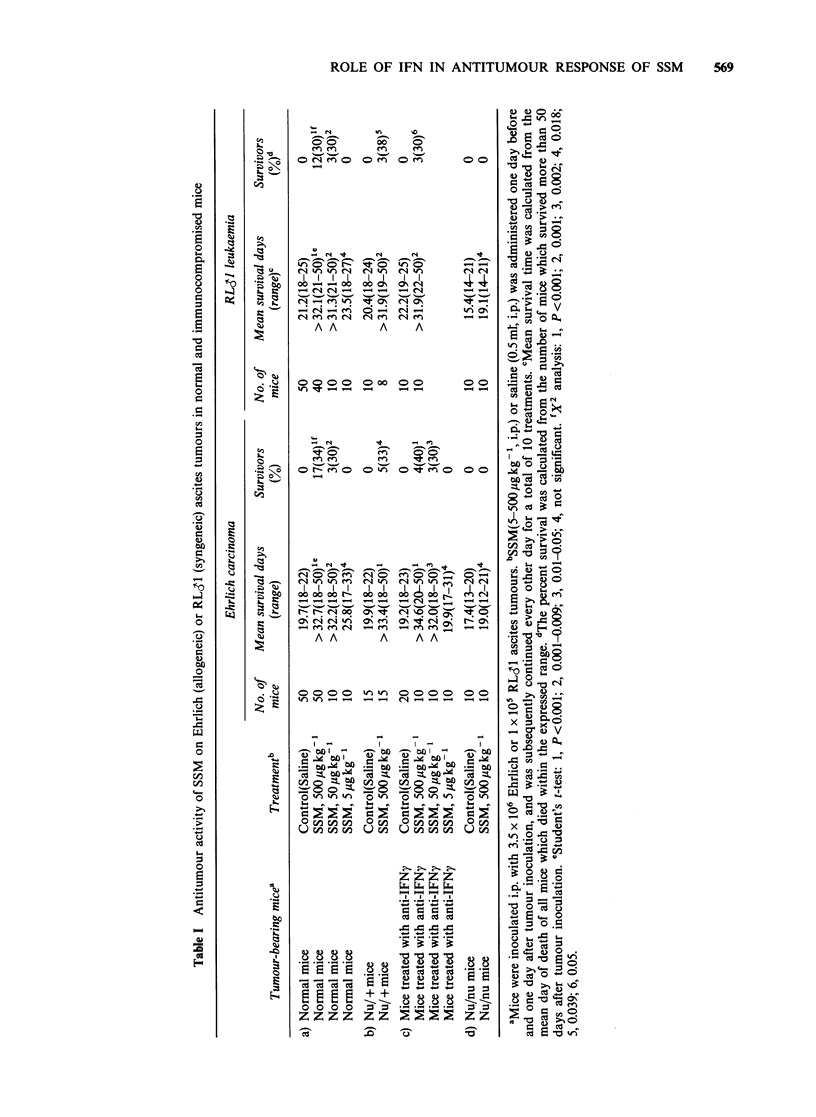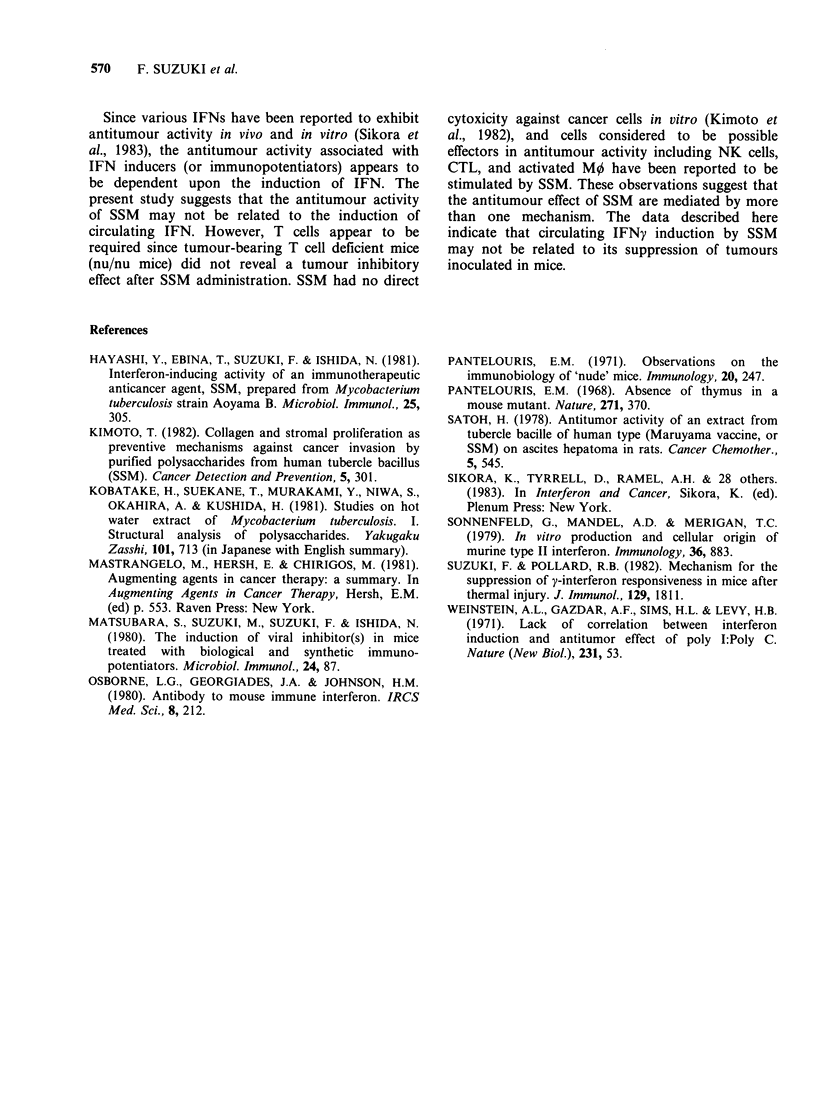# Lack of correlation between antitumour response and serum interferon levels in mice treated with SSM, an immunotherapeutic anticancer agent.

**DOI:** 10.1038/bjc.1986.89

**Published:** 1986-04

**Authors:** F. Suzuki, R. R. Brutkiewicz, R. B. Pollard


*Br. J. Cancer* (1986), **53**, 567–570

### Short Communication

### Lack of correlation between antitumour response and serum interferon levels in mice treated with SSM, an immunotherapeutic anticancer agent

F. Suzuki[1,2], R.R. Brutkiewicz[2] & R.B. Pollard[2]

[1]*Division of Infectious Diseases, Department of Internal Medicine and Department of Microbiology, University of Texas Medical Branch, Virology Division, Shriners Burns Institute, Galveston, Texas 77550, USA; and* [2]*Department of Microbiology, Kumamoto University Medical School, Kumamoto 860, Japan.*

Many immunomodulators induce the production of interferons (IFN) *in vivo* (Matsubara *et al.*, 1981) and exhibit antitumour activities in animals and patients with cancer (Mastrangelo *et al.*, 1981). The antitumour effect of IFN have been also demonstrated in experimental models and in various human tumours (Sikora *et al.*, 1983). These immunomodulators, as well as IFN, have been reported to increase natural killer (NK) cell activity, cytotoxic T-cell (CTL) activity, and macrophage (M$\phi$)-mediated tumour cell killing (Mastrangelo *et al.*, 1981). Despite a correlation between the *in vivo* antitumour activity of the agents and the ability to stimulate IFN production, exceptions have been reported (Weinstein *et al.*, 1971). SSM, an arabinomannanlipid preparation extracted from cultured cells of *Mycobacterium tuberculosis* strain Aoyama B (Kobatake *et al.*, 1981), is an immunopotentiating agent with IFN-inducing (Hayashi *et al.*, 1981) and antitumour activity (Satoh, 1978; Kimoto, 1982). The IFN induced by SSM has been shown to be gamma IFN (IFN$\gamma$) (Hayashi *et al.*, 1981). Since SSM did not display direct cytotoxicity against cancer cells when they were cultured with the agent (Kimoto, 1982), the antitumour effect of the agent might be expressed through host defence functions. In the present study, the role of SSM-induced circulating IFN$\gamma$ in the antitumour activity of the agent was investigated utilizing models of immunocompromised mice incapable of IFN production.

The *in vivo* strain of the BALB/c syngeneic leukaemia, RL♂1, was a gift from Dr J.Y. Djeu, Division of Virology, Bureau of Biologics, FDA, Bethesda, MD, USA. Ehrlich carcinoma cells adapted to mice from an *in vitro* strain, were obtained from the Department of Microbiology, University of Texas Medical Branch, Galveston, TX, USA. These tumours were maintained by serial passage *in vivo* by injecting $1\text{–}5 \times 10^6$ cells per mouse, i.p., in BALB/c mice. The SSM (lot No. 123, C-1) (Kobatake *et al.*, 1981) was kindly supplied by the Zeria Pharmaceutical Co., Ltd., Tokyo, Japan. SSM (5–500 $\mu g\,kg^{-1}$, i.p.) or saline (0.5 ml, i.p.) was administered one day before and one day after tumour inoculation, and was subsequently continued every other day for a total of 10 treatments. Two kinds of immunocompromised animals were used in this study: (i) Mice treated with anti-mouse IFN$\gamma$ antiserum which was prepared in rabbits immunized with staphylococcal enterotoxin A (SEA)-induced IFN$\gamma$. This material has been shown to preferentially inactivate the antiviral activity of IFN$\gamma$ *in vitro* while not reacting with IFN$\alpha$ or IFN$\beta$ (Osborne *et al.*, 1980). Since 5 $mg\,kg^{-1}$ of SSM administered i.v. to mice has been reported to result in peak IFN$\gamma$ titers of 320 $U\,ml^{-1}$ in the circulation (Hayashi *et al.*, 1981), a dose of this antiserum capable of neutralizing 2,500 $U\,kg^{-1}$ of IFN was given s.c. to mice immediately after administering SSM. (ii) Congenitally athymic nude (nu/nu) mice homozygous for the recessive gene (nu), which were severely limited in thymus-derived lymphocytes. These animals are deficient in Thy $1^+$ T lymphocytes (Pantelouris, 1968) which have been shown to produce IFN$\gamma$ (Sonnenfeld *et al.*, 1979). As a positive control, normal BALB/c mice were stimulated with SSM to induce IFN. Although haired littermates of the nude mice are heterozygous at the nu gene locus (nu/+), their T cell functions have been reported to be intact (Pantelouris, 1971). These nu/+ mice also served as positive controls. After inoculation of the tumour cells, mice were observed daily until day 50, and the percent survival was calculated from the number of mice which survived more than 50 days after tumour inoculation. For the determination of circulating IFN, blood samples were obtained from various mice at appropriate intervals after the injection of SSM. The blood samples were kept at 4°C overnight, then centrifuged at 1,250 *g* for

Correspondence: F. Suzuki.
Received 17 September 1985; & in revised form, 17 December 1985.

© The Macmillan Press Ltd., 1986

30 min, and obtained serum specimens were assayed for IFN on L-Galveston cells in a microplaque reduction assay as described previously (Suzuki & Pollard, 1982). To determine the dose response effect of SSM on IFN induction in various mice, sera were harvested 20 h after various doses of SSM administration. IFN levels were expressed as international $\mathrm{U ml^{-1}}$ as compared to the standard murine IFN (G-002-904-511). One U of the experimental IFN samples was equivalent to 1.2 U of the international standard.

As shown in Figure 1, although normal and nu/+ mice treated i.p. with $2.5\,\mathrm{mg\,kg^{-1}}$ of SSM had IFN in the circulation which peaked 20 h after SSM administration, normal mice treated with anti-mouse IFNγ antiserum and nu/nu mice stimulated with the same concentration of SSM had no detectable serum IFN activity at any of the time intervals examined. The impaired IFNγ responsiveness in immunocompromised mice treated with SSM was also observed following the administration of various concentrations of SSM. Sera were harvested 20 h after SSM injection and assayed. As shown in Figure 2, while doses of SSM $>170\,\mu\mathrm{g\,kg^{-1}}$ induced circulating IFN in normal and nu/+ mice, no IFN was detected in mice treated with anti-mouse IFNγ antiserum or nu/nu mice at any concentration used. This suggests that the ability to produce circulating IFN following stimulation with SSM was lacking in immunocompromised mice. Following determination of the IFN-inducing activity of SSM in normal mice,

**Figure 1** Interferon induction by SSM in normal and immunocompromised mice. Groups of 5 mice each (all of the BALB/c background) received one i.p. injection of $2.5\,\mathrm{mg\,kg^{-1}}$ of SSM and the sera obtained at 4 h intervals were assayed for IFN. The groups of mice utilized were as follows: mice treated with anti-mouse IFNγ antiserum (s.c., 2,500 neutralizing $\mathrm{U kg^{-1}}$, administered immediately after SSM treatment (-▲-), nu/+ mice (-△-), nu/nu mice (-■-), and normal BALB/c mice (-○-). The IFN titers were determined by the average obtained from 5 mice in each group.

**Figure 2** Effect of various concentrations of SSM on IFN induction in normal and immunocompromised mice. Five mice each (all of the BALB/c background) were treated i.p. with various concentrations of SSM ($5$–$2{,}500\,\mu\mathrm{g\,kg^{-1}}$). Sera from normal (-○-), nu/nu (-■-), nu/+ (-△-), or normal mice treated with anti-mouse IFNγ antiserum as described above (-▲-), were obtained from the mice 20 h after SSM administration and assayed for antiviral activity.

heterozygous littermates of nude mice and two kinds of immunocompromised mice, the antitumour activity of SSM in these groups of mice was investigated. As shown in Table Ia, significant antitumour activity was observed in normal mice bearing Ehrlich and RL♂1 ascites tumours when they were treated with both 500 and $50\,\mu\mathrm{g\,kg^{-1}}$ of SSM. However, when tumour-bearing normal mice were treated i.p. with $5\,\mu\mathrm{g\,kg^{-1}}$ of SSM or 0.5 ml of saline, all died. In addition, when nu/+ mice bearing tumours received $500\,\mu\mathrm{g\,kg^{-1}}$ of SSM, the antitumour effect was equivalent to that observed in normal mice (Table Ib). The antitumour activity of SSM was, therefore, detectable in this experimental tumour model and the following experiments were performed utilizing the two groups of immunocompromised mice. As shown in Table Ic, a significant protective effect was noted in tumour-bearing mice treated with anti-mouse IFNγ antiserum and 500 or $50\,\mu\mathrm{g\,kg^{-1}}$ of SSM as compared to those treated with saline. In T cell deficient (nu/nu) mice, however, no protective effect of SSM was observed possibly due to a deficit in supressor/cytotoxic Ly-2,3$^{+}$ T cells, which also produce IFNγ (Sonnenfeld *et al.*, 1979). As the tumour-bearing nu/nu mice had no inhibition of tumour growth following SSM administration, functional T cells, but not IFNγ induction, appeared to be required for the antitumour effect. In order to determine if serum IFN levels played a role during the treatment course with SSM, sera were obtained from all groups after each SSM administration and assayed for IFN activity. After several treatments with SSM, no serum IFN activity was detected in any of the groups (data not shown).

Since various IFNs have been reported to exhibit antitumour activity *in vivo* and *in vitro* (Sikora *et al.*, 1983), the antitumour activity associated with IFN inducers (or immunopotentiators) appears to be dependent upon the induction of IFN. The present study suggests that the antitumour activity of SSM may not be related to the induction of circulating IFN. However, T cells appear to be required since tumour-bearing T cell deficient mice (nu/nu mice) did not reveal a tumour inhibitory effect after SSM administration. SSM had no direct cytoxicity against cancer cells *in vitro* (Kimoto *et al.*, 1982), and cells considered to be possible effectors in antitumour activity including NK cells, CTL, and activated Mϕ have been reported to be stimulated by SSM. These observations suggest that the antitumour effect of SSM are mediated by more than one mechanism. The data described here indicate that circulating IFNγ induction by SSM may not be related to its suppression of tumours inoculated in mice.

### References

HAYASHI, Y., EBINA, T., SUZUKI, F. & ISHIDA, N. (1981). Interferon-inducing activity of an immunotherapeutic anticancer agent, SSM, prepared from *Mycobacterium tuberculosis* strain Aoyama B. *Microbiol. Immunol.*, **25**, 305.

KIMOTO, T. (1982). Collagen and stromal proliferation as preventive mechanisms against cancer invasion by purified polysaccharides from human tubercle bacillus (SSM). *Cancer Detection and Prevention*, **5**, 301.

KOBATAKE, H., SUEKANE, T., MURAKAMI, Y., NIWA, S., OKAHIRA, A. & KUSHIDA, H. (1981). Studies on hot water extract of *Mycobacterium tuberculosis*. I. Structural analysis of polysaccharides. *Yakugaku Zasshi*, **101**, 713 (in Japanese with English summary).

MASTRANGELO, M., HERSH, E. & CHIRIGOS, M. (1981). Augmenting agents in cancer therapy: a summary. In *Augmenting Agents in Cancer Therapy*, Hersh, E.M. (ed) p. 553. Raven Press: New York.

MATSUBARA, S., SUZUKI, M., SUZUKI, F. & ISHIDA, N. (1980). The induction of viral inhibitor(s) in mice treated with biological and synthetic immunopotentiators. *Microbiol. Immunol.*, **24**, 87.

OSBORNE, L.G., GEORGIADES, J.A. & JOHNSON, H.M. (1980). Antibody to mouse immune interferon. *IRCS Med. Sci.*, **8**, 212.

PANTELOURIS, E.M. (1971). Observations on the immunobiology of 'nude' mice. *Immunology*, **20**, 247.

PANTELOURIS, E.M. (1968). Absence of thymus in a mouse mutant. *Nature*, **271**, 370.

SATOH, H. (1978). Antitumor activity of an extract from tubercle bacille of human type (Maruyama vaccine, or SSM) on ascites hepatoma in rats. *Cancer Chemother.*, **5**, 545.

SIKORA, K., TYRRELL, D., RAMEL, A.H. & 28 others. (1983). In *Interferon and Cancer*, Sikora, K. (ed). Plenum Press: New York.

SONNENFELD, G., MANDEL, A.D. & MERIGAN, T.C. (1979). *In vitro* production and cellular origin of murine type II interferon. *Immunology*, **36**, 883.

SUZUKI, F. & POLLARD, R.B. (1982). Mechanism for the suppression of γ-interferon responsiveness in mice after thermal injury. *J. Immunol.*, **129**, 1811.

WEINSTEIN, A.L., GAZDAR, A.F., SIMS, H.L. & LEVY, H.B. (1971). Lack of correlation between interferon induction and antitumor effect of poly I:Poly C. *Nature* (*New Biol.*), **231**, 53.